# The Many Faces of Rap1 GTPase

**DOI:** 10.3390/ijms19102848

**Published:** 2018-09-20

**Authors:** Anna Jaśkiewicz, Beata Pająk, Arkadiusz Orzechowski

**Affiliations:** 1 Department of Physiological Sciences, Warsaw University of Life Sciences—SGGW, Nowoursynowska 159, 02-776 Warsaw, Poland; ancpatrin@gmail.com; 2 Independent Laboratory of Genetics and Molecular Biology, Kaczkowski Military Institute of Hygiene and Epidemiology, Kozielska 4, 01-163 Warsaw, Poland; bepaj@wp.pl

**Keywords:** Rap1 GTPase, guanine nucleotide exchange factors (GEFs), GTPase-activating proteins (GAPs), isoprenylation, geranylgeraniol

## Abstract

This review addresses the issue of the numerous roles played by Rap1 GTPase (guanosine triphosphatase) in different cell types, in terms of both physiology and pathology. It is one among a myriad of small G proteins with endogenous GTP-hydrolyzing activity that is considerably stimulated by posttranslational modifications (geranylgeranylation) or guanine nucleotide exchange factors (GEFs), and inhibited by GTPase-activating proteins (GAPs). Rap1 is a ubiquitous protein that plays an essential role in the control of metabolic processes, such as signal transduction from plasma membrane receptors, cytoskeleton rearrangements necessary for cell division, intracellular and substratum adhesion, as well as cell motility, which is needed for extravasation or fusion. We present several examples of how Rap1 affects cells and organs, pointing to possible molecular manipulations that could have application in the therapy of several diseases.

## 1. Introduction

Rap1 belongs to the Ras family of small molecular weight GTPases. The Ras subfamily is known to regulate many physiological responses including: cell adhesion, cell growth, apoptosis, cytoskeleton remodeling, motility, and intracellular vesicular transport [1]. Apart from controlling physiological processes, the Ras subfamily plays an active part in pathological processes. The Ras pathway is one of the most commonly deregulated pathways in human cancers. Activating mutations in *ras* genes occur in nearly 30% of all human malignant tumors [2]. These mutations typically render Ras constitutively GTP-bound, resulting in the activation of downstream effector pathways in the absence of extracellular stimuli [3]. Even if the *ras* gene mutation is absent, the loss of function or inactivation of the Ras GTPase-activating proteins (GAPs) or the upregulation of Ras guanine nucleotide exchange factors (GEFs) phenocopies activates mutations in the *ras* gene [2]. Rap1 has a highly similar amino acid sequence to Ras, pointing to the presence of interchangeable binding partners. Nevertheless, Rap1 also manifests opposing effects on cancer phenotypes [4].

The first report of the Rap1 protein was published in 1989, in which it was described as a Krev-1 protein with anti-oncogenic activity [5]. This was followed by another report that presented Rap1 as a Ras-related protein [6]. Despite numerous studies, the precise role of Rap1 has not been defined to date.

**Figure 1:**
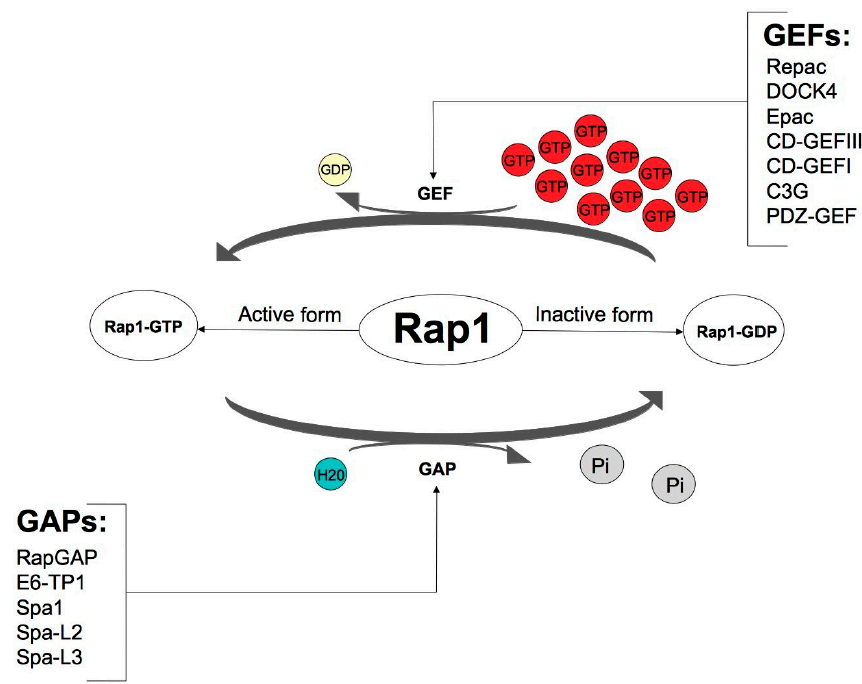
Control of Rap1 GTPase activity via guanine nucleotide exchange factors (GEFs) and GTPase-activating proteins (GAPs). Based on the KEGG Kanehisa Laboratories (https://www.kegg.jp/kegg-bin/show_pathway?map04015, accessed on 19 September 2018).

Although this protein is encoded by two different genes, Rap1 occurs in two isoforms: Rap1A and Rap1B, showing 95% identity [7]. Similarly to the other GTPases from the Ras subfamily, the Rap1 protein acts as a molecular switch by cycling between two states—an inactive GDP-bound form and an active GTP-bound form [8]. These modifications are strictly controlled by GEFs and GAPs. GEFs stimulate the replacement of GDP with GTP through the dissociation of GDP, thereby allowing abundant GTP to bind and activate Rap1. The inactivation of Rap1 is led by GAPs, which enhance intrinsic GTPase activity, resulting in GTP hydrolysis [9]. Because the intracellular concentration of free GTP vastly exceeds that of GDP in cells, nucleotide exchange on Ras increases the percentage of Ras-GTP and enhances the output. Signaling terminates when Ras-GTP is hydrolyzed to Ras-GDP. GAPs play an integral role in this process by stabilizing a transition state between Ras-GTP and Ras-GDP. This accelerates the half-life (T_1/2_) of the Ras GTPase from minutes to seconds [10] (Figure 1).

**Figure 2:**
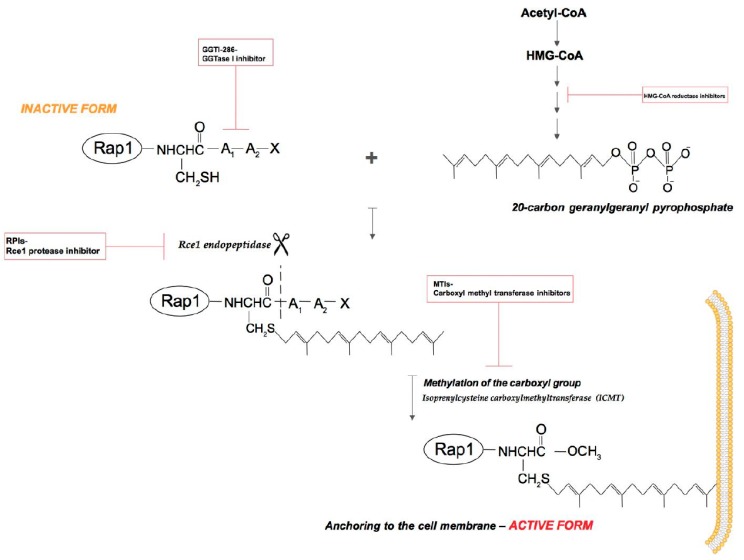
Rap1 GTPase isoprenylation. Rap1 post-translational modifications occurs in three steps. Step 1: the covalent attachment of a 20-carbon geranylgeranyl isoprenoid chain to the Cys residue in the CAAX (denoting the amino acid sequence Cys-aliphatic residue-aliphatic residue-X: usually Met, Ser, Gln or Leu) box located in the C-terminus (FTase and GGTase I but not GGTase II). Step 2: leavage off the three terminal amino acids via Rce1 endopeptidase (CAAX prenyl protease 2). Step 3: methylation of the isoprenylated Cys residue by the isoprenylcysteine carboxyl methyltransferase (ICMT) [13].

After the translation process, many proteins are incapable of action, they must undergo post-translational modifications. These modifications guarantee correct protein structure and dynamics [11]. A newly synthesized Rap1 protein like small GTPases, is a soluble cytosolic protein that must undergo isoprenylation to enable it to associate with appropriate lipid membranes [12] (Figure 2).

## 2. Activation of Rap1

Rap1 is activated in multiple signal transduction pathways depending on the cell type [14]. There is no common receptor for all types of cells whose stimulation would lead to the activation of the Rap1 protein. Rap1 is activated by the agonistic stimulation of various receptors coupled with tyrosine kinases or G protein-coupled receptors (serpentine receptors, GPCRs), including the thrombin receptor in platelets [15], the insulin receptor in ovary cells [16], the antigen receptor in lymphocytes [17], the high-affinity receptor for human granulocyte/macrophage colony-stimulating factor (GM-CSF receptor) and other serpentine receptors in neutrophils [18], and nerve cell growth factor receptor in PC12 cells [19].

At least three different Rap1-activated second messengers have been identified: 3′,5′ cyclic adenosine monophosphate (cAMP), calcium ion (Ca^2+^), and diacylglycerol (DAG) [20].

cAMP can inhibit or stimulate the Ras/mitogen-activated protein kinase (MAPK) pathway. There are also some similarities that have been identified between the mechanisms of action of Rap1A and cAMP, as Rap1A has a similar effect to Ras; it activates extracellular signal-regulated kinase 2 (MAPK ERK2) and induces cell proliferation and differentiation. The same effect occurs after an increase in the concentration of cAMP.

Some authors described Rap1 as being on a par with protein kinase A (PKA) in the activation of the serine/threonine kinase B-Raf. It has also been proven that Rap1 is able to activate various transcription factors and stimulate neuronal differentiation via an ERK-dependent, but Ras-independent pathway [21]. In addition, it has also been hypothesized that Rap1A may act as a cAMP-induced inhibitor in Ras signaling [22]. The outcomes of PKA activation are described in the Section 2.2.

cAMP is a high-ranking second messenger that, in eukaryotic cells, induces physiological responses ranging from growth, differentiation, and gene expression, to secretion, ion channel conduction, and neurotransmission [23]. Most of the above-mentioned effects have been attributed to the stimulation of PKA by cAMP [24], but cAMP-regulated GEFs (cAMP-GEFs) also bind cAMP and selectively activate the Rap1 protein in a PKA-independent manner. Two genes have been characterized by the presence of cAMP-binding motifs and Ras family GEF domains [25]. This suggests that the genes might code for cAMP-binding proteins that directly couple the cAMP signal transduction system to Ras family cascades and constitute cAMP-regulated GEF proteins (cAMP-GEFI and cAMP-GEFII). Two orthologs have been isolated from humans and rats: cAMP-GEFI and cAMP-GEFII. Some of physiological functions of cAMP may result from direct cAMP coupling to Rap1 effector pathways. Different levels of cAMP-GEF expression could confer cell type-specific cAMP regulation of Ras superfamily signaling related to growth and differentiation. Moreover, the *CalDAG-GEF* family genes link Ca^2+^ and DAG inputs to Ras-specific GEFs [25]. Additionally, Rap1 is among the few GTPases capable of recruiting GEFs for other GTPases (Vav2 for Rac, FRG for Cdc42) to the site where other GTPases are needed to act on actin cytoskeleton [26,27,28,29].

The second well-known Rap1 activator is Ca^2+^ [30]. The clearest role of calcium ions in the activation of Rap1 is observed in platelets, as Rap1 activation occurs independently of, and probably prior to, platelet aggregation and the association of Rap1 with the cytoskeleton [30].

However, PKC-dependent Rap1 activation has been described in neutrophils [18]. Interestingly, it turns out that both Ca^2+^ ionophore ionomycin and phorbol ester 12-*O*-tetradecanoylphorbol 13-acetate (TPA) are phosphatidylinositol phospholipase C beta (PLC-β) inductors, which produce elevated levels of intracellular free Ca^2+^ and DAG-mediated Rap1 activation. However, the inhibition of PLC-β and Ca^2+^ only marginally affect Rap1 activation, suggesting that additional pathways may control Rap1 activation [18].

As an example of cells in which the activity of Rap1 protein depends solely on DAG we can distinguish B cells. BCR and its involvement in Rap1 activation have been investigated. This process depends on the production of DAG by PLC-γ [31]. The activation of Rap1 by B cell antigen receptor (BCR) was greatly reduced in PLC-γ-deficient B cells, whereas both synthetic DAG and phorbol dibutyrate have been shown to activate Rap1 in B cells [31].

Completely different views can be drawn regarding the activation of Rap1 signaling in endocrine cells. Thyrotropin (TSH) stimulation of target cells is controlled by Rap1 activity and signaling upon cAMP and PKA. Rap1 is activated by TSH through a cAMP-mediated and PKA-independent mechanism. Apparently, Rap1 is blocked by phosphorylation in a PKA-dependent manner, since it is not inhibited by interference with PKA activity. Moreover, Rap1 activity is prolonged by PKA inhibitors, as is expression of Rap1A mutants lacking a PKA phosphorylation site. PKA exerts a negative effect on cAMP-stimulated Rap1 activity in some cells [32]. TSH is also able to stimulate Akt1 serine/threonine phosphorylation. Akt1 activity was shown to be considerably elevated by Rap1A, whereas it could not be evoked in cells expressing a putative dominant-negative Rap1A mutant. These findings reveal that the mechanisms of Rap1 regulation by cAMP is complex and includes PKA-independent activation and PKA-dependent negative feedback. Thus, cAMP and PKA-independent activation appear to be indispensable for TSH and Akt1 kinase signaling [32].

Another factor that triggers the activation of Rap1 in human HeLa cells is the adhesion of the cells to substratum. The opposite situation occurs in nonadherent hematopoietic mouse 32D cells in which Rap1 activation depends on the specific soluble agent G-CSF. However, both in human HeLa and mouse 32D cells, Rap1 is necessary for cell adhesion and probably for cell spreading [14]. Overexpression of the *spa-1* gene (signal-induced proliferation-associated *gene-1*) in both kinds of cells blocks cell adhesion and decreases the amount of Rap1 GTP in the adherent state, indicating that this gene can play an important role in the activation of Rap1 [14].

**Figure 3:**
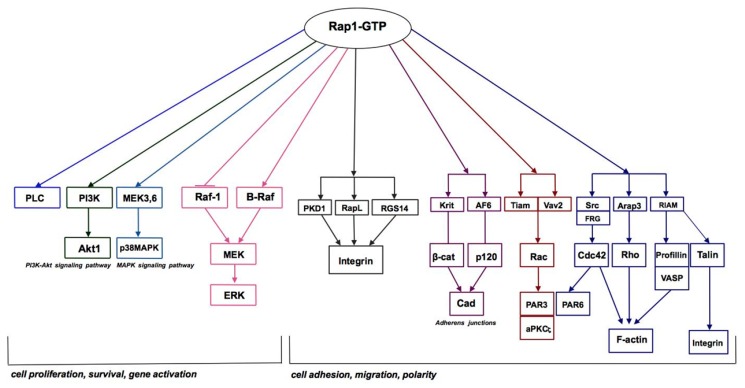
Rap1 GTPase signaling pathways. Based on the KEGG Kanehisa Laboratories (https://www.kegg.jp/kegg-bin/show_pathway?map04015, accessed on 19 September 2018).

According to results obtained from megakaryocytes, turbulence is also the mechanism responsible for the activation of Rap1 [33]. It was clearly demonstrated that a gentle mixing procedure is the only factor that is required to elevate the Rap1-GTP level, and this effect is completely independent of other factors that usually activate Rap1. A reasonable explanation for this phenomenon is that the cells subjected to the force tearing them apart from the substratum corresponds to increased binding for protection purposes [33]. In actuality, the description of how Rap1 is involved in blood vessel sensing in its environment is provided by the following elegant commentary: local changes in passage of proteins, fluid, and cells cause a response from the endothelial lining [34] (Figure 3). Several reports have described in detail how Rap1 contributes to the regulation of the endothelial barrier control in the blood vessels [35,36,37,38,39].

### 2.1. Isoprenylation

Isoprenylation is the basic molecular mechanism that is responsible for the activation of Rap1. Isoprenylation increases the hydrophobic characteristic of proteins, which ensures anchoring in cell membranes, and thus participation in signaling pathways [40]. This process relies on the covalent attachment of isoprenyl compounds such as farnesol or geranylgeraniol to the -SH group of the cysteine residue located at the C-terminus of the polypeptide chain or in its vicinity [41]. Isoprenylation is a necessary post-translational modification of many proteins involved in such diverse physiological processes, such as vision, regulation of blood vessel tension, cell proliferation, and bone tissue metabolism [41]. Increased interest in the protein isoprenylation process occurred in the 1980s and 1990s, when Ras proteins were found to be isoprenylated and mutations of genes encoding Ras proteins were found to often appear in cancer cells. Despite this, only 0.5–2% of intracellular proteins undergo isoprenylation [41], and the pharmacological inhibition of this process has considerable therapeutic potential. The most important course of research is the inhibition of isoprenylation of Ras proteins involved in the development of cancer. Moreover, GGTase I inhibitors could have a potential role in the treatment of cardiovascular diseases due to the involvement of Rho proteins in the regulation of NAD(P)H oxidase activity and secretion of nitric oxide (NO) [41].

We can distinguish two main trends in the pharmacological inhibition of protein isoprenylation: direct inhibition of protein prenyltransferases and inhibition of the synthesis of isoprenoids by bisphosphonates and statins as HMG CoAR inhibitors [41]. Statins reduce the concentration of all intermediates of mevalonic acid, thus blocking the process of farnesylation or geranylgeranylation. Rap1 is the only Ras family member protein whose activity depends exclusively on geranylgeraniol. This is in contrast to Ras (H-, K-, M-, and N-Ras), for which farnesylation with FTase is an important step in membrane localization, interaction, and GTPase activation [42]. However, the search for clinically useful FTase inhibitors as candidates for anticancer drugs has been disappointing [43].

The lens seems to be a good example of an organ in which such activation of the Rap1 plays a key role. Studies conducted on rat lens cell culture have shown that lovastatin induces changes in the characteristics of cataracts, that are prevented by geranylgeraniol but not farnesol [30]. These data may be indicative of the predominant role of Rap1 and its isoprenylation process in signaling pathways of ocular lens cells [44].

### 2.2. Other Post-Translational Modifications of Rap1

Distinct posttranslational modifications including GTPase phosphorylation lead to divergence in Rap1 activity, interactions with effectors, and cell signaling. Rap1 has been indicated as a strong candidate to contro**l** cell growth and differentiation through the cAMP-dependent activity of PKA and MAPK. Rap1A and Rap1B phosphorylations on serine 180 (S-180) and serine 179 (S-179), respectively, catalyzed by PKA, create binding sites necessary to contact kinase suppressor of Ras (KSR), the crucial regulator of the MAPK signaling pathway [45]. Although both Ras and Rap1 require phosphorylation for their ERK activation, in contrast to the Rap1-dependent effect that is amplified by PKA, the Ras-dependent effect is transient and rapidly terminated by this kinase. Thus, B-Raf binds to Rap1 independently of its Ras-binding domain, allowing Rap1 to couple to ERKs. Rap1 demands phosphorylation for its membrane localization and cell migration [46]. Phosphorylation of Rap1A and Rap1B leads to rather different consequences in regard to prenylation. Rap1A prenylation occurs independently of phosphorylations in the polybasic region (PBR). Phosphorylation on S-179 and S-180 of Rap1B inhibits binding to chaperone protein SmgGDS-607 and diminishes its prenylation and membrane localization [47]. PKA-dependent phosphorylations of Raf (S-259 for B-Raf, S-365 for C-Raf) were reported to inhibit signaling to Ras, even though these modifications do not affect Rap1 binding to Raf [45].

## 3. Rap1 Protein is A Link Between Cadherins and Integrins

Proper cell adhesion is the basis for maintaining the architecture of many tissues. Cell adhesion also plays a key role in the regulation of processes such as cell growth, differentiation, and migration. Cellular adhesion molecules (CAMs) mediate interactions between cells, and between cells and the extracellular matrix [48]. Under physiological conditions, the correct adhesion process is based on cell movement, intracellular communication, or signal transduction [49]. Conversely, impaired cell adhesion causes many pathological processes. E-cadherin-mediated cell-cell adhesion, for example, is lost during the development of most cancers [50]. When assessing the loss of intercellular connections, it is important to consider anoikis (“homeless” in Greek), a special type of programmed cell death that is observed in cells detached from the substratum and lacking integrin-mediated vital signals [51]. Cancer cells have the ability to resist anoikis and thus survive despite the loss of intracellular contacts [52]. Furthermore, this characteristic of tumor cells is correlated with reduced expression of cadherins. The loss of intercellular contacts due to a reduction in the amount of cadherins, the glycoproteins responsible for cell recognition, adhesion, and the strength of interactions between cells, creates favorable conditions for invasive cancer cells, enabling them to migrate [53].

There are many reports describing the role of Rap1 in cell adhesion and its involvement in different signaling pathways that are responsible for this process. An assessment of the influence of constitutively active Rap1A on the morphology of Ras-transformed MDCK-f3 cells showed that they are characterized by a non-colony forming, fibroblastoid phenotype with reduced E-cadherin mediated cell-cell contacts and did not form colonies [54]. It was observed that, due to the expression of constitutively active Rap1A, the formation of cadherin-mediated cell-cell contacts occurs and f3 cells acquire an epithelial phenotype. This transformation is Ras-independent, but the molecular mechanism through which Rap1A mediated these effects is not clear [54]. Furthermore, research conducted on cultured cells showed that the Rap1 plays a key role in the interactions between integrins and cadherins, which are responsible for proper tissue architecture. The authors found that the activity of the Rap1 protein is different in adherent and suspended cells and is significantly elevated upon cell detachment from the substratum. Upregulation of Rap1 activity occurred even before cell detachment from the substratum, as it was only caused by a breakdown of E-cadherin-mediated cell-cell junctions. They concluded that cell-cell adhesion has an inhibitory effect on Rap1. Thus, a decreased level of active Rap1 was associated with an increase in cell confluence, resulting in a greater number of intracellular interactions. However, the effect of cell adhesion on Rap1 activity was clearly independent of the integrin adhesive function but was entirely dependent on the formation of cell-cell contacts. It seems likely that Rap1 activity is a target of E-cadherin-mediated signaling events [55]. The authors also suggested that Rap1 is a possible downstream effector in signaling pathways involving sarcoma family protein tyrosine kinases (Src) activation through the disruption of E-cadherin-mediated cell adhesion [56].

## 4. Rap1 in Skeletal Muscle Differentiation

Skeletal muscle growth and regeneration (myogenesis) have two subsequent phases. Initially myoblasts proliferate, before withdrawing from the cell cycle, differentiating and fusing to form multinucleated myotubes and muscle fibers [56]. The exact position of Rap1 in each step is unknown, except for the fact that it accumulates during muscle cell differentiation [57]. Ultrastructural studies revealed that during skeletal myogenic differentiation, overexpression of the constitutively activated (GTP-bound) Rap1A protein inhibits myogenic differentiation, whereas the inactivated GDP-bound form of Rap1A favors myotube formation. It appears that a three-fold increase in the Rap1A protein is sufficient to inhibit differentiation [58]. Thus, the active Rap1A protein acts as a potent inhibitor of the differentiation program, which appears to contradict the fact that Rap1A protein accumulates during muscle cell differentiation [59,60]. The same group established that the various protein localizations are related to the GTP/GDP-bound state of Rap1 protein. In summary, these observations suggest that the Rap1A protein may regulate the morphological organization of the late endocytic compartments, and therefore, affects the intracellular degradation occurring during myogenic differentiation. The Rap1 protein has a specific intracellular location during mouse myogenic C2 cells differentiation. At the onset of cell fusion, transient accumulation of the GDP-bound Rap1A protein, referred as Rap1wt-GDP, is accompanied by concomitant perinuclear relocation at acidic organelles [58]. The Rap1A protein may regulate the intracellular locations of late endosomes and lysosomes, as do other Ras-related proteins [61]. Considering accounts that report on the accumulation of Rap1 protein in late endocytic vacuoles and lysosomes, it is assumed that the Rap1 protein actively participates in the autophagy process. Recent studies showed that autophagy is responsible for maintaining homeostasis during skeletal differentiation and growth [62]. Due to the higher energy requirements of mature myotubes compared to myoblasts, myotubes change the metabolic profile of mitochondria from anaerobic to aerobic glycolysis and the spatial rearrangement of the mitochondrial network. A more significant concentration of mitochondrial networks is found in mature myotubes compared to myoblasts. Due to this, myotubes are able to meet the energy requirements of mature and growing muscle cells.

### Rap1 Degradation in Skeletal Muscle Cells

Proteolytic enzymes present in late endosomes and lysosomes play key roles in the process of intracellular degradation and Rap1 has been shown to be responsible for regulation of these organelles [62]. Some reports showed that statins reduce the level of autophagy in muscle cell differentiation. Simultaneous treatment of myocytes with statins and geranylgeraniol restored the level of autophagy to almost control conditions. These results suggest a correlation between the expression of high-ranking Rap1 and muscle differentiation [63].

There is evidence that statin-induced muscle toxicity is associated with the inhibition of protein geranylgeranylation [63]. Geranylgeraniol was demonstrated to overcome stain-induced cytotoxicity [64]. Prenylation with non-sterol isoprenoids is an essential step in activating certain small GTPases including Rap1. This reaction is solely dependent on the geranylgeranylation of Rap1A catalyzed in skeletal muscle by prenyltransferases including protein geranyl transferase type I, EC 2.5.1.59, and probably, protein geranyl transferase type II, EC 2.5.1.60 [63].

Our latest research confirmed that Rap1 is indirectly targeted by statins that detain the availability of non-sterol isoprenoids in C2C12 muscle cells. Impaired prenylation of the Rap1 protein leads to lower muscle cell viability (repressed protein synthesis) and impeded autophagy. This mechanism might explain muscle damage and hampered myogenesis in statin-dependent myopathies [65].

Previous knowledge of the mechanisms that induce muscle atrophy indicates the key role of IGF-1/PI-3K/Akt1-pathway inhibition. The reduced Akt1 kinase activity observed upon statins treatment, resulted in an increase in dephosphorylation on FoxO3/FoxO1 transcription factors, their translocation into the nucleus, and activation of the *Atrogin1/MAFbx* gene encoding ubiquitin ligase E3, which eliminates MyoD, calcineurin, eIF3-F translation initiator, and FoxO1 [66]. Consequently, the regeneration and growth of skeletal muscle is inhibited. Our studies on C2C12 cells show that the Rap1, which becomes active due to prenylation with geranylgeraniol, plays a key role in the IGF-1/PI-3K/Akt1 signaling pathway [65].

## 5. Rap1 in the Inflammatory Response

According to some authors, the Rap1 protein is an important regulator of the inflammatory response [67]. Several functions of the Rap1 can be distinguished in the immune system, such as: (1) leukocyte motility, where activation of Rap1 is characterized by elevated levels of leukocyte movements; (2) antigen functioning, where Rap1 is responsible for the regulation of lymphocyte function-associated antigen 1 (LFA-1) activity for intercellular adhesion molecule 1 (ICAM-1); (3) leukocyte functioning, where Rap1 takes part in T cell receptor clustering and LFA-1 avidity for ICAM-1 following chemokine activation; (4) phagocytosis, where Rap1 is responsible for complement-mediated phagocytosis; (5) leukocyte transvascular migration, where Rap1 regulates integrin activation and extravasation on ICAM-1 and vascular cell adhesion protein 1 (VCAM-1); platelet endothelial cell adhesion molecule 1 (PECAM-1)-mediated leukocyte signaling; and (6) the functioning of endothelial immune cells, where Rap1 increases the number of cell junctions, which limits leukocyte transmigration [67].

Leukocytes, as the basic tool of the immune system, are responsible for the development of an inflammatory response, during which they undergo the following steps: rolling, activation, tight adhesion, and diapedesis [68]. Integrins are mainly responsible for the adhesion of leukocytes. Rap1 has been identified as a critical mediator of the reaction in which the cytoplasmic tail of CD31 (an important integrin adhesion amplifier); propagates signals that induce T cell adhesion via α4β1 (VLA-4) and β2 (LFA-1) integrins [69]. Thus, this study revealed that CD31 selectively activates Rap1, but not Ras, R-Ras, or Rap2. T cell adhesion to ICAM-1 was stimulated by an activated Rap1 mutant [69]. These data suggest a key role of Rap1 in the adhesion of leukocytes, which allows the inflammatory response to proceed.

Rap1 was also revealed as a target for the Toll-Like Receptors (TLRs) family, which play the most important role in the immune response. TLRs trigger both types of immune reactions, innate and adaptive, which work together to defeat infection. TLRs are the front-line defense for any microbial threat. In other words, these membrane receptors are of special scientific concern during any attempt to improve the treatment against infectious and inflammatory diseases [70]. TLRs are found in effector cells, such as T and B lymphocytes, dendritic cells, macrophages, and epithelial cells. Currently, several (at least 10) different TLRs have been identified, each having sufficient specificity to distinct bacterial, fungal, and viral components [71]. In addition, some data has indicated that acute stimulation of TLR2 and TLR5 leads to Rap1 activation in THP-1 myelomonocytes [72]. Activated Rap1 has been discerned as early as 1 min after human monocytic cell line 1 (THP-1) cell input with the TLR2 ligand. Moreover, a study that focused on the exposure to the mAB24-recognized immunogenic epitope showed that THP-1 cells with the r*ap1A* gene silenced with siRNA cannot make use of TLR2 and TLR5-induced cell adhesion to ICAM-1. Consequently, TLR2- and TLR5-triggered stimulation of β2-integrin was abolished. Transfection with siRNA to inhibit the *rap1a* gene, however, does not affect β2-integrin cell membrane expression in THP-1 cells. Rac1-mediated (Ras-related C3 botulinum toxin substrate 1), Nox2 (NADPH oxidase 2), and reactive oxygen species (ROS) formation after TLR2 and TLR5 ligation increase Rap1 activity. Accordingly, Rac1 improves β2-integrin affinity, thus promoting leukocyte adhesion. Therefore, Rac1 is considered to be a central player in TLR-mediated immune responses. In turn, TLR-mediated Nox2-dependent ROS generation is involved in antimicrobial killing, cytokine release, and control of cell death pathways in white blood cells [72].

Another example of Rap1 activity in the immune system was described when T cells deficient in Rap1 were demonstrated to have significantly elevated ERK and p38 kinase activity in comparison to T cells of wild type mice [73]. Moreover, in a hypersensitivity model of Rap1-deficient mice, reduced contact dermatosis was observed compared to wild type mice. This points to the in vivo impact of Rap1-deficiency on the inflammatory response [73].

Furthermore, an analysis of the MAP kinase activation status in CD3+ cells after different types of stimulation showed an increase in the activation of p38 and JNK in *rap1A^−^/^−^* T cells as compared to wild type cells. Rap1A-deficient T cells are also characterized by a stronger secretion of the interleukin (IL)-9, IL-5, IL-6, and IL-10. These findings suggest that the proper immunological response requires Rap1 protein activity [73].

## 6. Rap1 in Neoplastic Transformation

Now we address the dark side of Rap1, which is connected with its involvement in cell motility and the cell adhesion of transformed cells. It is widely known that the ability of cells to move beyond their normal physiological location is crucial in the formation of cancer metastases. The loss of epithelial polarity and consequent disruptions in the tissue architecture, a hallmark of aberrant differentiation, are early features of various malignancies [74]. A good example is the involvement of Rap1 in the regulation of tissue polarity, lumen formation, and invasive potential in human breast cancer cells [75].

Further, it was confirmed that Ras GTPases play significant roles in the development of numerous aspects of the malignant phenotype by promoting cell cycle progression, resistance to apoptotic stimuli, neovascularization and tumor cell motility, invasiveness, and metastasis [76]. Consequently, small GTPases are appealing targets for the development of cancer chemotherapeutic agents [77]. Ras oncoproteins are difficult targets for pharmacological interventions due to their lack of protein binding pockets or allosteric regulation [4]. A potential strategy is to directly target the Ras and Rho proteins by inhibiting post-translational modifications through blocking farnesylation or geranylgeranylation (FTase and GGTase I inhibitors), blocking HMG-CoA reductase with statins, blocking the digestion of the three carboxyl terminal amino acids of the CAAX box by the endopeptidases Rce1, or preventing Ras or Rho proteins from achieving or maintaining the active GTP-bound state by guanine-mimetic analogues. Targeting Ras and Rho signaling pathways has become a major endeavor in the fight against cancer [77].

Protein isoprenylation is catalyzed by three enzymes (protein prenyltransferases); FTase, GGTase I, and RABGGTase or GGTase-II [78]. FTase and GGTase I do not show absolute specificity to their substrates and FTase can bind geranylgeranyl diphosphate (GGPP) with 15-fold lower affinity than farnesyl pyrophosphate (FPP), whereas GGTase I binds FPP with approximately 300 times lower affinity than GGPP [41]. Considering the fact that the activity of Rap1 depends solely on GGTase I [79], the use of inhibitors of this enzyme may be effective in treating cancers in which the activity of Rap1 is crucial. Moreover, we can assume that pharmacological inhibitors of GGTase I may be a fairly safe group of compounds due to their very low affinity to FPP. Therefore, the wide effect of inhibiting the isoprenylation of proteins that play significant roles in physiological processes will not arise (Figure 2.)

Previous promising results showed that the blocking of Rap1 activation could prevent the metastasis of melanoma cells in vivo in a B16F1 mouse model [80]. It was also noticed that in vivo Rap1 activation is necessary for tumor cells that have been anchored to endothelial cells, which further promotes transendothelial migration (TEM). Reported findings suggest that Rap1 may be a new target that can be used to limit the spread of metastatic cells that leave the vasculature via an active TEM [81].

Furthermore, in breast cancer cells, knockdown of junctional adhesion molecule-A (JAM-A), whose expression was shown to minimally influence total Rap1 expression but significantly reduce the active form of Rap1 protein [82]. Moreover, the authors found that the Rap1 pharmacological inhibitor GGTI-298 reduced MCF7 cancer cell migration in a scratch-wound assay [81]. A similar effect was observed after treatment with an inhibitory antibody targeting β1-integrin. It was shown that the combined treatment of MCF7 cells with inhibitors of Rap1, β1-integrin, and JAM-A resulted in decreased cancer cell migration, from approximately 35% to 18% in a wound closure assay after six hours. This indicates that JAM-A, Rap1, and β1-integrin are likely to function together in a linear signaling pathway in breast cancer cells [81]. The authors hypothesized that JAM-A is a possible upstream effector for Rap1 and β1-integrin in breast cancer that is crucial for cell adhesion, migration, and invasion [81].

In prostate cancer cells, Rap1 activation is also connected to its metastatic potential. It was shown that the activation of Rap1 increases prostate cancer (CaP) cell migration and invasion through the α4, β3, and αvβ3 integrins [82]. By extending the effect of Rap1 activity in CaP metastasis in vivo, the introduction of activated Rap1 into CaP cells was shown to dramatically enhance the rate and incidence of CaP metastasis in a xenograft mouse model [82]. In addition, the Rap1 protein was demonstrated to be directly suppressed by miR-203, which is a short non-coding RNA molecule that is supposed to be a tumor suppressor gene [83]. The inhibition of Rap1 activity on this pathway contributes to the inhibition of prostate cancer cell metastasis [83].

On the other hand, the regulation of Rap1 function was described in a prostate cancer model engagement of p120 catenin (p120ctn) [84]. p120ctn is an armadillo repeat family member and a component of the cadherin-catenin complex in the adherens junctions. Based on its subcellular localization, it has pleiotropic functions, such as modulation of the turnover rate of membrane-bound cadherins, regulation of the activation of Rho GTPases in the cytoplasm, and modulation of nuclear transcription. p120ctn is an Src substrate that can be phosphorylated at different tyrosine, serine, and threonine residues, and can dock various kinases and phosphatases [84]. p120ctn has also been postulated to regulate Rap1 activity. In human DU145 prostate cancer cells, p120ctn depletion was associated with increased Rap1 activity [85]. A reduced level of E-cadherin increases Rap1 activity and thus, triggers the disruption of adherens junctions, which promotes metastasis [85].

The Rap1 protein has also been linked with the development of leukemia. *Spa-1* has been described as a principal Rap1 GTPase-activating protein in hematopoietic progenitors. Spa-1-deficient mice developed a spectrum of myeloid disorders that resemble chronic and blast crisis phase human myelogenous leukemia (CML) and myelodysplastic syndrome, as well as anemia. Overexpression of an active form of the Rap1 protein was shown to promote the proliferation of normal hematopoietic progenitors, whereas Spa-1 overexpression markedly suppressed it. Furthermore, restoring the s*pa-1* gene in a Spa-1-deficient leukemic blast cell line resulted in the dissolution of Rap1 GTP accumulation and concomitant loss of the leukemogenicity in vivo. These data suggest that Rap1 has a crucial role in myeloproliferative stem cell disorders and Spa-1 has a tumor suppressor function [86].

Rap1 may also be a potential target for impeding the metastatic progression of tumors that exit the vasculature via epithelial-mesenchymal transition (EMT) [80]. The blocking of Rap1 activation and expression of a constitutively active form of Rap1 reduced the ability of mouse B16F1 melanoma cells to extravasate from the microvasculate and formed metastatic lesions in the lungs [80]. This was correlated with a decreased ability of the tumor cells to undergo EMT in vitro and form dynamic, F-actin-rich pseudopodia that penetrate capillary endothelial walls in vivo [80].

Rap1GAP was identified as a putative tumor suppressor gene in pancreatic cancer where studies showed that expression of Rap1GAP was significantly downregulated [87]. In vitro and in vivo studies showed that loss of Rap1GAP promotes pancreatic cancer growth, survival, and invasion, and may function through modulation of integrin activity. Overexpression of Rap1GAP blocks Rap1 activation, and as a result, inhibits cell proliferation and survival. Moreover, Rap1GAP-overexpressing pancreatic cancer cells treated with apoptosis-inducing chemotherapeutic drugs, such as 5-FU and etoposide, induced up to 28% (5-FU) and 13% (etoposide) higher rates of apoptosis in comparison to control cells. These results suggest that the loss of Rap1GAP expression in human pancreatic adenocarcinoma may render cells resistant to apoptosis and, therefore, play a role not only in tumor progression but also in chemotherapeutic resistance [87].

## 7. Rap1 in Genetic Disorders

There is no consistent theory that would allow us to place the Rap1 on a specific signaling pathway. However, it seems likely that this protein plays an important role in controlling mammalian target of rapamycin complex 1 (mTORC1) kinase, which is responsible for maintaining the balance between the anabolic and catabolic processes in cells. Many recent studies have focused on the roles of GTPases in various pathologies associated with abnormal gene function.

A study of tumor-suppressor genes used *tsc1* and *tsc2* as subjects to investigate whether the Rap1 could be a link between the hamartin-tuberin complex (TSC1-TSC2) and mTORC1. Their activity was shown to indirectly affect the mTORC1 and thus, regulate cell growth. Abnormalities involving these two genes causes a disease entity called tuberous sclerosis (TSC), a human genetic disease involving the development of benign tumors as well as rare malignancies in a variety of tissues [88].

Tuberous sclerosis stems from a loss of function mutation in genes at two different loci. The first is the tuberous sclerosis-2 gene (*tsc2*), that encrypts an open reading frame (ORF) with a putative protein product of 1784 amino acids (Tsc2). The Tsc2 product (tuberin) encompasses a sequence of amino acids at the homologous C-terminus to the catalytic domain of Rap1GAP. As demonstrated by an immunoprecipitation study, the activity of native tuberin specifically stimulates the intrinsic GTPase activity of Rap1A. However, tuberin does not stimulate the GTPase activity of Rap2, Ha-Ras, Rac, or Rho. These data suggest that loss of the tuberin function leads to constitutive activation of Rap1 in tumors in patients with tuberous sclerosis. Rap1A has also been suggested to be a tuberin substrate for GAP activity in vitro, but the magnitude of its action is weak [89]. In any case, defective tuberin with a loss of GAP activity might cause constitutive activation of Rap1A or other GTPases, which, in turn could lead to deregulated mitogenic signaling. This theoretical model would be analogous to the role of mutant neurofibromin in modulating Ras GTPase activity in schwannoma [90].

The TSC1-TSC2 complex seems to be a GAP for Rap1 in the stimulation of mTORC1, although the Rheb protein is the best described and best known studied target of the tuberin-hamartin complex [91].

Another genetic disorder that is possibly connected to Rap1 function is Kabuki syndrome (KS). KS is a part of the “RASopathies”—a group of rare genetic diseases caused by gene mutations of the Ras-MAPK pathway [76]. Any aberrancy of this signaling pathway has serious consequences for development and can cause a number of different syndromes, including: Kabuki syndrome, Cardio-Facio-Cutaneous syndrome (CFC), Costello syndrome (CS), Legius syndrome (LS), Neurofibromatosis type 1 (NF1), Noonan syndrome (NS), as well as Noonan syndrome with multiple lentigines (NSML). These listed syndromes, as a group, are quite rare, whereas the RASopathies are the most frequently occurring genetic conditions [92].

KS is caused by de novo dominant germline mutations in KMT2D or KDM6A [93]. A link between Rap1A and Rap1B to KS and identification of the KMT2D-containing ASCOM complex as a major regulator of the MEK/ERK pathway, have been demonstrated, providing insight into the molecular mechanisms underlying KS [93]. The anatomical and cellular findings further suggest that Rap1 and KMT2D coordinate cell tissue embedment through the function of polarized actin and myosin IIa. KS can have opposite effects, either on the activation or silencing of the MAPK pathway, depending on whether the Rap1 proteins operate through B-Raf or Raf1, which may have a direct impact on many aspects of this disease [93,94].

## 8. Summary and Perspectives

Three decades of research have significantly expanded our knowledge about the Rap1 protein. Rap1, a central regulator of cell adhesion and motility, appears to play a key role in most cellular pathways. It is impossible to unambiguously determine the direct actions of Rap1. The opposite effects of Rap1 activation are the result of its participation in various cell signaling pathways in various cell types. Even in normal, pathologically unchanged cells, Rap1 has different effects depending on the cell type (Table 1).

For example, overexpression of Rap1 in Swiss 3T3 cells induces DNA synthesis [106], but introduction of constitutively activated Rap1 in astrocytes [107] and in keratinocytes [108] inhibits cell proliferation. In myocytes, the correct prenylation of Rap1 determines the viability of the cells [65]. It is also difficult to clearly define the role of Rap1 in intercellular interactions. Rap1 activity is essential for the adhesion of HeLa and 32D cells [14]. In f3 cells, Rap1 promotes the formation of intercellular links and contributes to the change in their phenotype from fibroblastoid to epithelial - mesenchymal-epithelial transition (MET) [54]. On the contrary, it has also been demonstrated that intercellular adhesion inhibits Rap1 activity [55], whereas turbulence is the mechanism to maintain Rap1 activity in megakaryocytes [33]. Also, in the immune system, increased activity of the Rap1 protein is positively correlated with lymphocyte movement [67], but surprisingly, in endothelial immune cells, Rap1 is responsible for proper cell junction formation [69]. However, the observations from cancer cells support the hypothesis that Rap1 activity is crucial for cell movement [75,77,78,79,80,81,82,83,85]. The silencing of Rap1 expression inhibits the metastasis of cancer cells, as measured via the scratch-wound assay test and through the use of GGTI-298, a Rap1 pharmacological inhibitor [78]. It seems that the activity of the Rap1 protein is a prerequisite for the functioning of most cell types, and the direction of action of this GTPase results from the functions performed by each cell type.

Disturbances in the functioning of this GTPase are reflected in the pathophysiology of almost all body systems, as this functioning determines the development of a number of genetic diseases [80,90]. Rap1 protein seems to be also key to ensuring the architecture of the epithelium via promoting cadherin-mediated cell adhesion, as it is a guarantee of proper tissue architecture [54,55]. It is also responsible for the opposite phenomenon, the EMT process, which occurs during embryonic development or in pathological states where cell division and differentiation are disrupted [84].

The disorder in the signaling pathway of Rap1, in many cases, promotes cancer progression. Targeting signaling of Rap1 could be a method of controlling the development of cancer and metastasis. During the development of tumors, changes in the amount of total Rap1 protein are not as important as changes in the amount of Rap1 GAPs. Unfortunately, in vitro studies have shown large discrepancies between particular types of cancer; in some types of cancer, the loss of Rap1 GAPs favors the malignancy of cancer, and in other cases, it inhibits progression. However, the blocking of Rap1 activity may be valuable in the future, especially in oncology, due to the fact that it is the only GTPase whose prenylation is exclusively dependent on geranylgeraniol. The remaining GTPases deficiencies in geranylgeranylation can be compensated by farnesylation.

Moreover, studies on muscle atrophy induction by statins suggest that the combination of statin therapy with geranylgeraniol could eliminate the side effects of statins on skeletal muscle. At the same time, the fact that Rap1 is involved in the development of many cancer types suggests that the potential pharmacological use of geranylgeraniol for supplementation with statin therapy needs further study.

Rap1 protein constantly cycles between its active form, which is associated with GTP, and the inactive form, which is associated with GDP. It seems that this protein should be treated as a molecular switch that coordinates the functioning of various cell pathways. Two forms, active and inactive, regulate intracellular processes as well as intercellular interactions, thus conditioning the proper functioning of tissues. However, considering the fact that Rap1 protein is present in many cellular metabolic processes, targeting Rap1 GTPase could be a turning point in the fight against many diseases.

## Figures and Tables

**Table 1:** The Rap1 effectors and consequences of their activation, respectively.

Rap1 GTPase Effector	Cell Type	Effect	References
**Afadin/AF-6**	Epithelial cells	Adherens junction formation	[95,96]
**Arap3**	Endothelial cells	Regulation of lamellipodia formation	[97]
**IQGAP1**	Epithelial cells	Regulation of cytoskeleton fomation	[98]
**Krit-1/CCM1**	Arterial/Venous endhothelial cells	Maintaining the integrity of endothelial junctions	[99]
**Mst1** **RAPL**	Leukocytes	Integrin α1β2 activation	[100]
**Phg2 serine/threonine protein kinase**	*Dictyostelium discoideum* cells	Regulation of chemotaxis and cell adhesion	[98]
**PKD1 serine/threonine protein kinase D1**	T cells	Promotion of cell adhesion	[101]
**Rac** **Cdc42**	MDCK	Actin cytoskeleton assembly	[27]
**Radil**	Neural crest (NC) cells	Promotion of integrin-mediated cell adhesion and migration	[102]
**RA-RhoGAP**	Neuronal cells	Regulation of neurite outgrowth	[103]
**RIAM**	Hematopoetic cells	Talin dependent integrins activation	[104]
**Rasip1/Rain**	Endothelial cells	Maintaining proper endothelial barrier functioning	[105]
**Raf-1 serine/threonine protein kinase** *(the Rap1 GTPase inhibitory effect)* B-Raf **serine/threonine protein kinase** *(the Rap1 GTPase activating effect)*	PC12 cells	Neuronal differentiation	[21]
**Tiam1** **Vav2**	Fibroblasts	Promotion of cell spreading	[26]

## Abbreviations

5-FU	Fluorouracil
AF6	Afadin
Akt1	RAC-alpha protein serine/threonine kinase
aPKCζ	Atypical protein kinase C zeta
Arap3	Arf-GAP with Rho-domain, ANK repeat and PH domain-containing protein 3
BCR	B cell antigen receptor
B-Raf	B-Raf proto-oncogene protein serine/threonine kinase, MAPKKK
C3G	Rap guanine nucleotide exchange factor 1
Ca^2+^	Calcium ion
CAAX	Cys-aliphatic residue-aliphatic residue-X
CAD	Cadherin 1, type 1, E-cadherin
CalDAG-GEF	Calcium/diacylglycerol guanine nucleotide exchange factor
cAMP	3’,5’ cyclic adenosine monophosphate
CAMs	Cellular adhesion molecules
Cdc42	Cell division control protein 42
CD-GEFII	Ras guanyl-releasing protein 2
CD-GEFIII	Ras guanyl-releasing protein 3
CFC	Cardio-Facio-Cutaneous syndrome
CLM	Chronic myelogenous leukemia
CS	Costello syndrome
C3G	Rap guanine nucleotide exchange factor 1
DAG	Diacylglycerol
DOCK4	Dedicator of cytokinesis protein 4
E6TP1	Signal-induced proliferation-associated 1 like protein 1
eIF3-F	Eukaryotic translation initiation factor 3 subunit F
EMT	Epithelial-mesenchymal transition
ERK1/2	Extracellular signal-regulated kinase 1/2, MAPK
F-actin	Filamentous actin
FOXO1	Forkhead box protein O1
FOXO3	Forkhead box protein O3
FPP	Farnesyl pyrophosphate
FRG	Ferm, RhoGEF and pleckstrin domain protein 2
FTase	Farnesyl transferase
GAPs	GTPase-activating proteins
G-CSF	Granulocyte colony stimulating agent
GEFs	Guanine nucleotide exchange factors
GGPP	Geranylgeranyl diphosphate
GGTase I	Geranylgeranyl transferase-1
GGTase II	Rab geranylgeranyl transferase, geranylgeranyl transferase-2
GM-CSF	High-affinity receptor for human granulocyte/macrophage colony-stimulating factor
GPCRs	G protein-coupled receptors, serpentine receptors
GTPase	Guanosine triphosphatase
HMG-CoAR	3-hydroxy-3-methylglutaryl coenzyme A reductase
ICAM-1	Intercellular adhesion molecule 1
ICMT	Isoprenylcysteine carboxyl methyltransferase
IGF-1	Insulin like growth factor
JAM-A	Junctional adhesion molecule-A
JNK	C-Jun N-terminal kinase
KDM6A	(K)-specific demethylase 6A
KMT2D	Lysine-specific methyltransferase 2D
Krit	Krev interaction trapped protein 1
KS	Kabuki syndrome
KSR	Kinase Suppressor of Ras
LFA-1	Lymphocyte function-associated antigen 1
LS	Legius syndrome
MAFbx	Muscle-specific F-box
MAPK	Mitogen-activated protein kinase
MDCK-f3	Mesenchymal Ras-transformed Madin-Darby canine kidney cells
MEK	Mitogen-activated protein kinase, MAPKK
MEK3,6	Mitogen-activated protein kinase 3/6, MAPKK
MET	Mesenchymal-epithelial transition
miR-203	Short non-coding RNA molecule
mTORC1	Mammalian target of rapamycin complex 1
NF1	Neurofibromatosis type 1
NO	Nitric oxide
Nox2	NADH oxidase 2
NS	Noonan syndrome
NSML	Noonan syndrome with multiple lentigins syndrome
p120ctn	Catenin (cadherin-associated protein), delta 1
p38MAPK	P38 MAP kinase
PAR3	Partitioning defective protein 3
PAR6	Partitioning defective protein 6
PECAM-1	Platelet endothelial cell adhesion molecule—also known as CD31—cluster of differentiation 31
PI3K	Phosphatidylinositol-4,5-bisphosphate 3-kinase catalytic subunit alpha/beta/delta
PKA	Protein kinase A
PKD1	Protein kinase D1
PLC	Phosphatidylinositol phospholipase C
PLC-β	Phospholipase C beta
PLC-γ	Phospholipase C gamma
PDZ-GEF	Rap guanine nucleotide exchange factor 2
Rac1/RAC	Rac1-Ras-related C3 botulinum toxin substrate 1
Raf-1	B-Raf proto-oncogene protein serine/threonine kinase, MAPKKK
RapL	Ras associated domain-containing protein 5
RapGAP1	Rap1 GTPase activating protein 1
Repac-RAPGEF5	Rap guanine nucleotide exchange factor 5
RGS14	Regulator of G-protein signaling 14
RIAM	Amyloid beta A4 precursor protein-binding family B member 1- interacting protein
Rho	Ras homolog gene family, member A
Spa1	Signal-induced proliferation-associated *gene 1*
Spa-L2	Signal-induced proliferation-associated 1 like protein 2
Spa-L3	Signal-induced proliferation-associated 1 like protein 3
Src	Sarcoma family protein tyrosine kinases
TEM	Transendothelial migration
THP-1	Human monocytic cell line 1
TIAM	T-lymphoma invasion and metastasis-inducing protein 1
TLN	Talin
TLRs	Toll-Like Receptors
TPA	12-*O*-tetradecanoylphorbol 13-acetate
TSC	Tuberous sclerosis
TSC1-TSC2	Hamartin-tuberin complex
TSH	Thyrotropin
VASP	Vasodilator-stimulated phosphoprotein
Vav2	Vav guanine nucleotide exchange factor 2
VCAM-1	Vascular cell adhesion protein 1
α4β1 (VLA-4)	Very late antigen-4
β-cat	Catenin beta 1
